# GABAergic ventrolateral preoptic projection to dorsomedial hypothalamus recapitulates post-ischemic neuroprotection by hypothermia

**DOI:** 10.1038/s41419-026-08536-0

**Published:** 2026-03-10

**Authors:** Pelin Dilsiz, Aysenur Ozpinar, Buse Balaban, Halil İbrahim Koç, Serdar Altunay, Saltuk Buğra Baltacı, Fatma Zehra Hapil, Thorsten Roland Doeppner, Egor Dzyubenko, Mustafa Çağlar Beker, Dirk Matthias Hermann, Ertuğrul Kılıç

**Affiliations:** 1 Department of Molecular Biology and Genetics, Faculty of Science and Letters, Istanbul Technical University, Istanbul, Türkiye; 2 Research Institute for Health Sciences and Technologies (SABITA), Istanbul Medipol University, Istanbul, Türkiye; 3 Department of Physiology, School of Medicine, Istanbul Medipol University, Istanbul, Türkiye; 4 Graduate School of Health Sciences, Istanbul Medipol University, Istanbul, Türkiye; 5 Department of Medical Biology, School of Medicine, Istanbul Medeniyet University, Istanbul, Türkiye; 6 Department of Medical Biology, Institute of Health Sciences, University of Health Sciences, Istanbul, Türkiye; 7 Department of Medical Biology, Faculty of Medicine, Akdeniz University, Antalya, Türkiye; 8 University Medical Center Göttingen, Department of Neurology, Göttingen, Germany; 9 Klinikum Emden, Department of Neurology, Emden, Germany; 10 Department of Neurology, University Hospital Essen, University of Duisburg-Essen, Essen, Germany; 11 Department of Physiology, School of Medicine, Istanbul Medeniyet University, Istanbul, Türkiye

**Keywords:** Cell death in the nervous system, Trauma, Cellular neuroscience

## Abstract

Therapeutic hypothermia by exogenous cooling induces potent neuroprotection. Post-stroke, therapeutic hypothermia so far did not translate into clinically applicable therapies due to hypothermia-associated side-effects compromising patient outcome. The hypothalamus contains two major thermoregulatory centers in the ventrolateral preoptic area (vlPOA) and dorsomedial hypothalamus (DMH), which are connected via gamma-aminobutyric acid (GABA)-ergic fibers. Using chemogenetic and optogenetic approaches, we explored the role of this GABAergic projection in regulating body temperature responses, cerebral blood flow, and ischemic injury in *Vgat-cre* mice exposed to transient middle cerebral artery occlusion (MCAo). Using a chemogenetic approach, we show that the inhibition of a set of GABAergic DMH^VGAT^ neurons, which under physiological conditions induces hyperthermia, is essential to drive hypothermia, which decreases cerebral blood flow post-MCAo and protects against ischemic reperfusion injury via mechanisms involving preservation of astrocytic homeostatic functions. This phenotype is recapitulated by the optogenetic activation of the GABAergic vlPOA^VGAT^ neurons, which similarly induces hypothermia and protects against ischemic injury. The GABAergic vlPOA^VGAT^ DMH pathway provides a potent target for neuroprotective therapies. We hypothesize that modulating central temperature responses via this pathway may not elicit the undesirable side effects associated with exogenous brain cooling.

**Figure:**
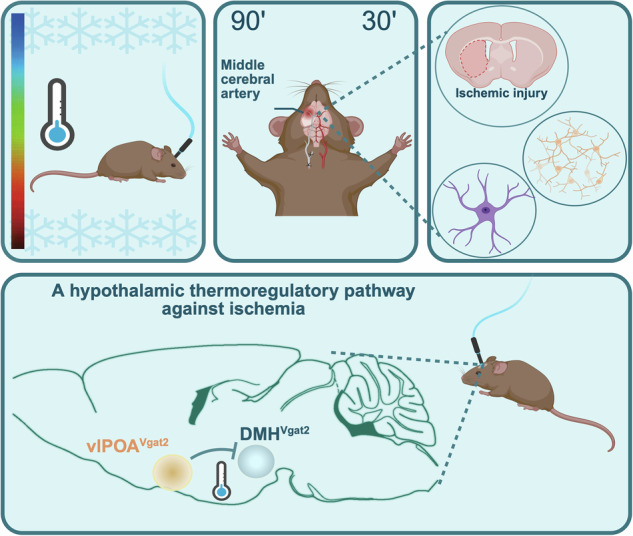
**Thumbnail: Graphical abstract:** GABAergic vlPOA^VGAT^ → DMH pathway activation lowers body core temperature, limits post-ischemic infarct volume, and enhances neuronal survival by reducing reperfusion damage. Hypothermia was chemogenetically or optogenetically induced in mice exposed to 90 or 30 min middle cerebral artery occlusion (MCAo). Structural and functional consequences of GABAergic vlPOA^VGAT^ → DMH pathway modulation were assessed.

**Thumbnail: Graphical abstract:** GABAergic vlPOA^VGAT^ → DMH pathway activation lowers body core temperature, limits post-ischemic infarct volume, and enhances neuronal survival by reducing reperfusion damage. Hypothermia was chemogenetically or optogenetically induced in mice exposed to 90 or 30 min middle cerebral artery occlusion (MCAo). Structural and functional consequences of GABAergic vlPOA^VGAT^ → DMH pathway modulation were assessed.

## Introduction

Body temperature homeostasis is strictly regulated through thermoregulatory centers in the preoptic area and hypothalamus, which receive peripheral thermal signals and integrate them with endocrine, autonomic, and behavioral information [1, 2], providing a homeostatic feedback that maintains core body temperature (*T*_core_) in a narrow range in homeothermic species [3–5]. Albeit the maintenance of *T*_core_ is crucial for various physiological functions, reducing *T*_core_ has broad clinical benefits under conditions of brain ischemia and trauma, since it promotes the survival of damaged brain tissue [6, 7]. Therapeutic hypothermia induced by exogenous cooling potently decreases ischemic injury in experimental stroke models by regulating regional cerebral blood flow (CBF), oxidative stress, and inflammation, in addition to maintaining blood-brain barrier (BBB) integrity [8–12].

Ischemic stroke is an eminent global health burden, which affects 1 in every 4 adults over the age of 25 [13], with increasing prevalence due to rising life expectancy [14, 15]. Therapeutic hypothermia has been proposed to promote post-ischemic brain tissue survival in human patients [16–18] and improve the neurological outcome of comatose subjects [19, 20]. However, therapeutic hypothermia induced by exogenous or intraarterial cooling did not promote stroke outcome in randomized clinical trials, which was due to undesirable side effects, namely cardiac rhythm disturbances, hypokalemia, coagulopathy and infections, which outweighed the neuroprotective actions of hypothermia, preventing clinical patient recovery [21–25]. The quest for therapeutic strategies that enhance stroke outcome raises the need for treatments capable to induce neuroprotection in human stroke settings. Since several of the side effects above are likely associated with exogenous or intraarterial cooling, we here explored neural circuits in the hypothalamus that mediate hypothermia post-ischemia, aiming to define if these circuits might be suitable as a neuroprotective target.

Recently, a subset of dorsomedial hypothalamus (DMH) neurons expressing gamma-aminobutyric acid (GABA) was shown to receive robust input from GABAergic ventrolateral preoptic area (vlPOA) and regulate thermal information in mammals [26]. Upon deactivation, these DMH^VGAT^ neurons were found to induce hypothermia, which was also detected when the nerve terminals of GABAergic vlPOA neurons in the same brain region were activated [26]. DMH-projecting vlPOA^VGAT^ neurons suppressed the thermogenic effects of DMH^VGAT^ neurons, which indicated the significance of the vlPOA^VGAT^ → DMH pathway in thermoregulation [26]. The involvement of this pathway in post-ischemic neuroprotection was so far unknown. Given its central role in thermal information processing, we hypothesized that DMH-projecting GABAergic vlPOA^VGAT^ neurons promote neuroprotection in the ischemic brain by inducing hypothermia. Herein, we demonstrate that chemogenetic inhibition of DMH^VGAT^ neurons and optogenetic activation of the vlPOA^VGAT^ → DMH projection decreases *T*_core_ significantly, which in turn facilitates neurological recovery after focal cerebral ischemia evidenced by reduced infarct volume and neuronal reperfusion injury. The vlPOA^VGAT^ → DMH pathway provides a promising target for neuroprotection therapies.

## Materials and methods

### Legal aspects

This study has been conducted under the ethics standards of the EU Guidelines on the Care and Use of Laboratory Animals (Directive 2010/63/EU) in agreement with Turkish National guidelines for animal experimentation. The study has been approved by local animal experimentation authorities (Animal Research Ethics Committee, Istanbul Medipol University; reference number: 01/12/2021-75).

### Animal holding and study blinding

Experimental procedures are reported in accordance with ARRIVE guidelines (Animal Research: Reporting of In Vivo Experiments). Male Cre-recombinase expressing *Vgat-cre* mice *(Slc32a1*^*tm2(cre)Lowl*^/J, stock 016962; Jackson Labs, Bar Harbor, ME, USA) back-crossed with C57Bl/6 mice (Stock 000664; Jackson Labs) were housed in a 12-h light (07:00–19:00) and dark (19:00–07:00) cycle at 22–24 °C ambient temperature, having ad libitum access to standard food and water. At the age of 8–12 weeks (20–25 g), mice were randomly assigned to experimental groups by sequential allocation, ensuring balanced animal numbers on each surgery day. Experimenters were blinded to group allocation at all stages of the study (animal experiments, histochemical stainings, and data analysis). Animal welfare was monitored daily throughout the study. Mice exhibiting a body weight reduction following adeno-associated virus (AAV) vector injection to below 20 g at the time of MCAo were excluded from the study.

### Statistical planning

Statistical planning was done by a sample size calculator (https://clincalc.com/stats/samplesize.aspx). Assuming an alpha error of 5% and a beta error (1–statistical power) of 20%, these calculations determined that 8 animals were needed per group for behavioral and histochemical analyses, provided that the mean value was modified by 35% and that the standard deviation of the data sample was 25% of the mean value (effect size: 1.4). To account for animal dropouts, 10 animals per group were stereotactically injected throughout the study.

### Adeno-associated virus (AAV) production

Recombinant AAV production was performed as described previously [27]. Cre-dependent recombinant AAV vectors purchased from http://www.addgene.org/ were used as follows: *rAAV2/1-CAG-DIO-GFP* (10^14^ vg/mL)*, rAAV2/1-EF1a-DIO-hM4D(Gi)-mCherry* (4 × 10^12^ vg/mL) and *rAAV2/1-EF1a-DIO-hChR2(H134R)-EYFP-WPRE-HGHpA* (1.8 × 10^14^ vg/mL) (*CAG: cytomegalovirus (CMV) enhancer/chicken beta-actin promoter, EF1a*: *human elongation factor-1 alpha* promoter, *DIO*: *double-floxed inverted open-reading frame*).

### Stereotactic viral injections and optical fiber implantation

Viral injections were performed as described previously [28–30]. Briefly, P30-P40 mice were anesthetized with 1% isoflurane (30% O_2_, 70% N_2_O) and placed in a stereotactic instrument (David Kopf Instruments, Tujunga, CA, USA). After a skin incision, the animals’ skull was exposed and trephinated using a small drill for stereotactic injection. A total of 600 nl virus was injected intracranially to each side using a pulled glass pipette (Wiretrol; Drummond Scientific, Broomall, PA, USA) with a 50 μm tip diameter. Injections were performed into the DMH (bregma: −1.50 mm, midline: ±0.35 mm, dorsal surface: −4.90 mm) and vlPOA (bregma: −0.10 mm, midline: ±0.70 mm, dorsal surface: −5.60 mm) at a rate of 30 nL/min using a micromanipulator (Narishige, East Meadow, NY, USA), allowing 10 min for each injection. For in vivo photostimulation, a ferrule-capped optical fiber (200 μm core diameter, NA = 0.50, ThorLabs) was implanted above the DMH (bregma: −1.50 mm, midline: ±0.35 mm, dorsal surface: −4.40 mm) after viral injections. The ferrules were fixed with dental cement, and the mice were allowed to recover for at least 3 weeks.

### Clozapine injection and photostimulation

For chemogenetic stimulation, 0.3 mg/kg clozapine (Tocris Bioscience, Bristol, UK) or normal saline was intraperitoneally (i.p.) administered to DMH^VGAT:hM4D^ designer receptors exclusively activated by designer drugs (DREADD) mice or DMH^VGAT:GFP^ non-DREADD control mice [30] obtained by rAAV-mediated transduction. In vivo photostimulation of DMH-projecting vlPOA^VGAT^ neurons was performed using a 473 nm diode laser (Doric Lenses Inc., Quebec City, Canada) through a ferule-capped optical fiber implanted in vlPOA^VGAT:ChR2^ or vlPOA^VGAT:GFP^ mice [30] obtained by rAAV-mediated transduction using the following stimulation protocol: 10 Hz stimuli with 10 ms pulse length were applied for 1 s; this stimulus was repeated every 4 s [30]. Optical stimulation was started 30 min before MCAO and continued until the end of MCAO, i.e., onset of reperfusion.

## Behavioral studies

### Body temperature measurement

Body core temperature (*T*_core_) was recorded using a thermocouple rectal probe and thermometer (Physitemp Instruments, Clifton, NJ, USA). *T*_core_ of DMH^VGAT:hM4D^ or DMH^VGAT:GFP^ mice was measured for 30 min at baseline. Then, 0.3 mg/kg clozapine was administered, and *T*_core_ was recorded for 3 h. In the meantime, *T*_core_ was measured using an infrared fusion thermal camera (Ti90; Fluke, Everett, WA, USA). Snapshot images were taken at the specified time points, and an average of 3 recordings was obtained. Similarly, *T*_core_ was recorded using the thermocouple rectal probe in vlPOA^VGAT:ChR2^ or vlPOA^VGAT:GFP^ mice during a 30 min baseline recording and subsequent 2 h photostimulation. *T*_core_ was also assessed using the infrared fusion thermal camera. Snapshot images were obtained.

### Neurological score

In mice exposed to 90 min MCAo, neurological deficits were evaluated using the 5-point Bederson score at 24 h after reperfusion. In this score, 0 reflects normal function, 1 reflects flexion of the torso and contralateral forelimb upon lifting the animal by the tail, 2 reflects circling to the contralateral side but normal posture at rest, 3 reflects reclination to the contralateral side at rest, and 4 reflects absence of spontaneous motor activity.

### Open field test

Following a post-operative recovery period, mice exposed to 30 min MCAo were transferred to the testing room and single-housed in Coulbourn Habitest cages (Coulbourn Instruments, Allentown, PA, USA) in cotton bedding without being handled for 3 days. The investigator then handled the mice for 3 days to reduce stress. The circular test chamber, having a 100 cm diameter and surrounded by a 35 cm tall sidewall, was divided into three sections, including an outer wall zone (17.7% of diameter, close to the wall), an intermediate transition zone (32.3% of diameter), and an inner zone (50% of diameter, the center of the chamber) [31, 32]. On the test days, animals were placed in an open field test chamber 30 min after clozapine or saline administration and observed for 10 min. The assay was traced with a CCF camera and blindly analyzed by two researchers using ANY-maze software (Version 4.99; Stoelting Co., Dublin, Ireland).

### Focal cerebral ischemia induction

Focal cerebral ischemia was induced by intraluminal middle cerebral artery occlusion (MCAo) as described previously [33]. To initiate chemogenetic inhibition of DMH^VGAT^ neurons, 0.3 mg/kg clozapine was intraperitoneally administered to DMH^VGAT:hM4D^ or DMH^VGAT:GFP^ mice 30 min before MCAo, whereas optogenetic activation of vlPOA^VGAT^ → DMH fibers was provided via photostimulation starting with MCAo until animal sacrifice. Mice were deeply anesthetized with 1% isoflurane (30% O_2_, remainder N_2_O), and their *T*_core_ was monitored using a feedback-controlled heating system (Harvard Apparatus, Holliston, MA, USA). CBF was measured and monitored by Laser Doppler flow (LDF) measurement using a flexible 0.5 mm fiber optic probe (PIMSoft; Perimed, Järfälla, Sweden). The optic probe was attached to the skull above the core of the middle cerebral artery territory (2 mm posterior/6 mm lateral from the bregma). Following a neck incision, the left common and external carotid arteries were isolated and ligated and a microvascular clip (FE691; Aesculap, Tuttlingen, Germany) was temporally placed on the internal carotid artery. A 7.0 silicon-coated nylon monofilament (701934PK5Re; Doccol, Sharon, MA, USA) was subsequently inserted into the common carotid artery through a narrow incision and advanced 9 mm distal to the carotid bifurcation for MCAo.

Ninety or 30 min after the onset of ischemia, reperfusion was induced by monofilament removal. At 24 h after reperfusion, mice exposed to 90 min MCAo were decapitated under deep anesthesia. The brains were collected and immediately frozen on dry ice. A total of 18 μm-thick coronal sections were obtained using a cryostat (CM1950; Leica, Wetzlar, Germany) and subsequently used to analyze infarct size, brain edema, and IgG extravasation. Mice exposed to 30 min MCAo were transcardially perfused in deep anesthesia at 72 h after reperfusion using 0.1 M 0.1 M pH 7.4 phosphate-buffered saline (PBS) followed by 4% paraformaldehyde (PFA) in 0.1 M pH 7.4 PBS. Brains were harvested, post-fixed in the same fixative for 4 h, and transferred to a 30% sucrose solution overnight. A total of 75 μm brain sections were collected with a vibratome (VT1000S; Leica) for immunohistochemistry.

### Laser speckle imaging

CBF was also evaluated by laser speckle imaging in DMH^VGAT:hM4D^ or DMH^VGAT:GFP^ mice intraperitoneally treated with clozapine (0.3 mg/kg). Anaesthetized mice were placed in a stereotactic instrument. A CCD camera was located approximately 10 cm above the brain to detect CBF variations using a Pericam PSI System (PIMSoft; Perimed) using a 785 nm wavelength laser, which penetrates approximately 500 μm deep into the brain surface. Speckle images were collected every 2 s with 20 μm spatial image resolution. The regional CBF was recorded for 90 min, the mean CBF was calculated by blood perfusion imaging software (PIMsoft; Perimed), and changes in relative CBF were calculated.

### Evaluation of infarct volume, brain edema, and serum IgG extravasation

Infarct volume and brain edema were evaluated at millimeter intervals across the forebrain in animals exposed to 90 min MCAo. Brain sections were stained with cresyl violet [34]. ImageJ software (National Institute of Health, Bethesda, MD, USA) was used for infarct measurements, outlining healthy tissue in both hemispheres, which were subtracted from each other for infarct area measurement. Infarct volume was obtained by integrating infarct areas at various rostro-caudal brain levels. Brain edema was calculated by subtracting areas of the contralateral hemisphere from areas of the ischemic hemisphere at the level of the bregma (which is the core of the middle cerebral artery territory) and dividing the resulting values by the corresponding areas of the contralateral hemisphere. Infarct volume was defined as the primary outcome of this study in mice exposed to 90 min MCAo, whereas brain edema and all subsequently listed readouts were regarded as secondary outcomes.

In 90 min MCAo mice, brain sections collected from the bregma level were rinsed with 0.1 M PBS to remove intravascular IgG, fixed in 4% PFA and blocked with methanol/ 0.3% H_2_O_2_. Sections were then immersed in 5% bovine serum albumin (BSA) and normal swine serum (1:1000) in 0.1 M PBS and incubated for 1 h in biotinylated goat anti-mouse IgG (sc-2013; Santa Cruz Biotechnology, Santa Cruz, CA, USA). After staining with an avidin peroxidase kit (Vectastain Elite; Vector Labs, Newark, CA, USA) and diaminobenzidine (Sigma-Aldrich, St. Louis, MO, USA), IgG extravasation in the ischemic striatum was analyzed densitometrically using ImageJ software. The optical densities of corresponding contralateral non-ischemic tissue were subtracted from those in the ischemic tissue for background staining correction.

### Immunohistochemistry and microscopy

Brain sections obtained from the level of the bregma of 30 min MCAo mice were blocked in 0.1 M PBS containing 0.1 mM Triton-X100 (PBS-T) and 5% normal goat serum for 1 h at room temperature. Sections were subsequently immersed in blocking solution containing monoclonal mouse anti-pyruvate kinase M2 (PKM2) (1:300; sc-365684; Santa Cruz Biotechnology), monoclonal rabbit anti-Bcl-xL (1:500; 2764; Cell Signaling, Danvers, MA, USA), monoclonal rat anti-GFAP (1:500; 13-0300; Thermo Fisher Scientific, Waltham, MA, USA), polyclonal rabbit anti-c-Fos (1:000; 2250; Santa Cruz Biotechnology), Alexa Fluor 488-conjugated monoclonal mouse anti-NeuN (1:100; MAB377; Merck-Millipore, Burlington, MA, USA), or Alexa Fluor 555-conjugated monoclonal mouse anti-GFAP (1:100; 3656; Cell Signaling) antibodies. After an overnight incubation at 4 °C, brain sections were rinsed with PBST. Sections exposed to non-conjugated primary antibodies were then exposed to Alexa Fluor 488-conjugated secondary antibody (1:500; A11001; goat anti-mouse IgG (H + L); Invitrogen, Waltham, MA, USA), Alexa Fluor 488-conjugated secondary antibody (1:500; A11008; goat anti-rabbit IgG (H + L), Invitrogen) Alexa Fluor 568-conjugated secondary antibody (1:500; A11011; goat anti-rabbit IgG (H + L), Invitrogen) or Alexa Fluor 594-conjugated secondary antibody (1:500; A11007; goat anti-rat IgG (H + L), Invitrogen) for 1 h at room temperature. Brain sections were again rinsed with PBST. Sections were counterstained with 4′, 6-diamidino-2-phenylindole (DAPI) and mounted with Fluoromount (F4680; Sigma-Aldrich). Additional brain sections were labeled with terminal transferase dUTP nick end labeling (TUNEL) kit (In Situ Cell Death Detection Kit; Roche, Basel, Switzerland) to detect neuronal injury through DNA fragmentation. Imaging was performed by confocal microscopy (Carl Zeiss, Jena, Germany). Nine different regions of interest (ROIs) from the striatum, each measuring 62,500 μm^2^, were evaluated to detect neuronal survival and injury. NeuN^+^ cells or DNA-fragmented cells were analyzed in the ischemic and contralateral striatum. Neuronal survival was evaluated by dividing the density of NeuN^+^ surviving neurons in both hemispheres. Astrocytic responses were evaluated by measuring the total area of GFAP immunoreactivity in the ischemic hemisphere. To measure PKM2 and Bcl-xL expression levels in astrocytes and non-astrocytic cells, binary masks representing GFAP^+^ astrocytes were generated by manual thresholding in ImageJ Software. Average signal intensities were measured within (for astrocytes) and outside of (non-astrocytic cells) the masks. Neuronal survival was defined as the primary outcome of this study in mice exposed to 30 min MCAo, whereas all other readouts were considered as secondary outcomes.

### Statistical analysis

All results were shown as mean ± SEM values. Differences between the two groups were tested by two-tailed unpaired Student’s *t* tests after confirming normal distribution using Shapiro-Wilk tests. Non-normally distributed data were evaluated using Mann–Whitney *U* tests (Statistical Table 1). *p* values were calculated using Prism 9.5 (GraphPad Software Inc.). Outlier tests were removed only when data deviated more than two standard deviations from mean values. *p* values < 0.05 were considered as statistically significant.

## Results

### Chemogenetic silencing of GABAergic DMH^VGAT^ neurons induces hypothermia

**Fig. 1:**
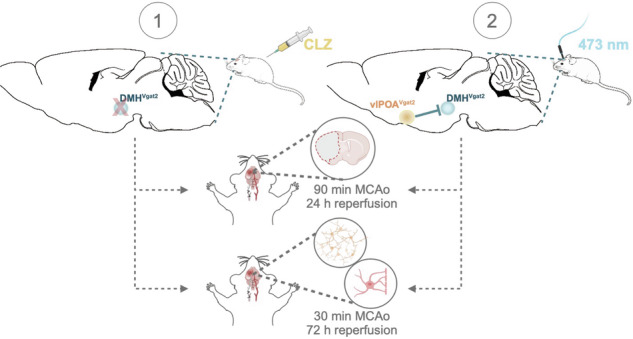
Schematic illustration of the experimental design. Hypothermia was induced by chemogenetic silencing of dorsomedial hypothalamic DMH^VGAT^ neurons by clozapine (CLZ) or optogenetic activation of the GABAergic ventrolateral preoptic area vlPOA^VGAT^ → DMH pathway by 473 nm laser light. Consequences for brain tissue survival and ischemic injury were investigated.

**Fig. 2:**
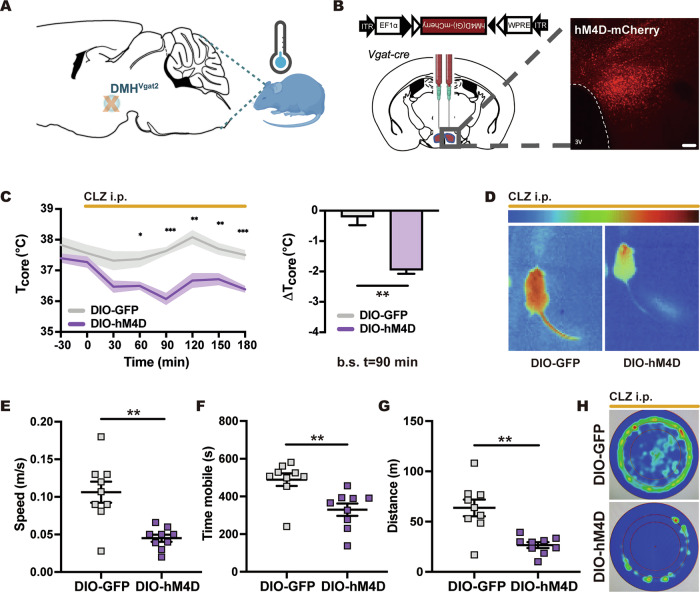
Chemogenetic silencing of GABAergic DMH^VGAT^ neurons induces hypothermia. **A** Cartoon illustrating chemogenetic DMH^VGAT^ neuron silencing as a strategy for inducing hypothermia. **B** An AAV vector with double-floxed inverted open-reading frame (DIO) hM4D(Gi) was bilaterally injected into the DMH of *Vgat-cre* mice, resulting in selective expression of the DREADD hM4D(Gi) in GABAergic DMH^VGAT^ neurons. Representative photomicrograph showing DMH^VGAT:hM4D^ expression. Scale bar: 500 μm. **C** Core body temperature measurements of DMH^VGAT:hM4D^ and DMH^VGAT:GFP^ mice (*n* = 9 each) after intraperitoneal (i.p.) clozapine (CLZ) delivery at *t* = 0 (left panel). Maximum body temperature changes were observed at *t* = 90 min. **D** Infrared thermography demonstrating decreased body temperature and tail vasoconstriction upon DMH^VGAT^ neuron inhibition. Open field tests revealed that **E** average speed, **F** time mobile, and **G** total distance covered decreased upon CLZ injection in DMH^VGAT:hM4D^ compared to DMH^VGAT:GFP^ mice (*n* = 9 each). **H** Cumulative heatmaps depicting time spent in different locations of the open field chamber during DMH^VGAT^ silencing. Note that DMH^VGAT^ silencing decreased overall animal motor activity. Data are mean ± SEM values. *p < 0.05, **p < 0.01, ***p < 0.001.

GABAergic neurons residing in the DMH were previously shown to drive hypothermia upon inhibition and promote thermogenesis when activated [26]. To gain insight into their role in central body temperature control, we stereotactically administered a Cre-dependent *rAAV2/1-EF1a-DIO-hM4D(Gi)-mCherry* in the DMH of *Vgat-cre* mice (Fig. 1). Chemogenic inhibition of GABAergic DMH^VGAT^ neurons by clozapine (Fig. 2A, B) significantly decreased core body temperature by 1.95 ± 0.11 °C in DIO-hM4D DREADD mice compared to mice receiving a non-DREADD (DIO-GFP) control vector, confirming the involvement of DMH^VGAT^ neurons in *T*_*core*_ regulation (Fig. 2C, D). Furthermore, we showed that locomotor activity decreased upon the chemogenetic silencing of DMH^VGAT^ neurons (Fig. 2E–H), which represents a homeostatic response towards temperature changes. Upon careful inspection, we did not observe any behavioral abnormalities upon chemogenetic DMH^VGAT^ silencing in this study. Our data argue in favor of a specific thermoregulatory response.

### DMH^VGAT^ silencing reduces infarct volume, neurological deficits, and brain edema

**Fig. 3:**
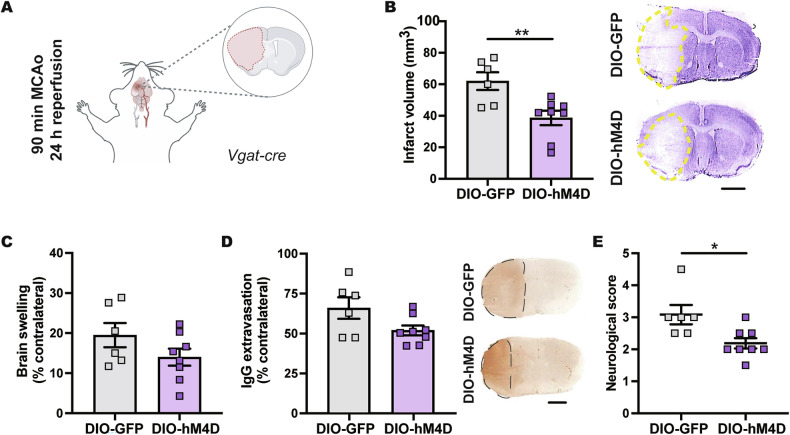
Chemogenetic DMH^VGAT^ silencing decreases infarct volume, neurological deficits and brain edema. **A** Cartoon showing the 90 min MCAo model followed by 24 h reperfusion that results in focal infarcts of the striatum and overlying cortex. Effect of CLZ-induced DMH^VGAT^ silencing on **B** infarct volume and **C** brain swelling examined by cresyl violet staining and **D** BBB leakage measured by serum IgG extravasation analysis and **E** neurological deficits evaluated by the Bederson score in DMH^VGAT:hM4D^ DREADD mice (*n* = 8) compared to DMH^VGAT:GFP^ non-DREADD control mice (*n* = 6). Representative cresyl violet and IgG extravasation images are shown. Scale bars: 2 mm. Data are mean ± SEM values. *p < 0.05, **p < 0.01.

Considering that chemogenetic silencing of GABAergic DMH^VGAT^ neurons reduced body temperature, we next investigated whether DMH^VGAT^ silencing induces neuroprotection after ischemic stroke induced by 90 min MCAo (Fig. 3A and Supplementary Fig. S1A), which was earlier shown to induce focal infarcts in the striatum and the overlying cortex, in addition to moderate brain edema of the ischemic hemisphere compared to the contralateral hemisphere [34–36]. Chemogenetic DMH^VGAT^ silencing significantly reduced infarct volume in DIO-hM4D mice at 24 h post-MCAo (Fig. 3B). Similarly, chemogenetic DMH^VGAT^ silencing reduced brain swelling (Fig. 3C) and BBB leakage measured by IgG extravasation (Fig. 3D), although the latter effects lacked to show statistical significance. Neurological deficits examined by the Bederson score were significantly reduced by DMH^VGAT^ silencing (Fig. 3E). Taken together, our findings demonstrate that DMH^VGAT^ silencing robustly improved neurological outcome after stroke.

### DMH^VGAT^ silencing increases post-ischemic neuronal survival and preserves the astrocytic homeostatic state

**Fig. 4:**
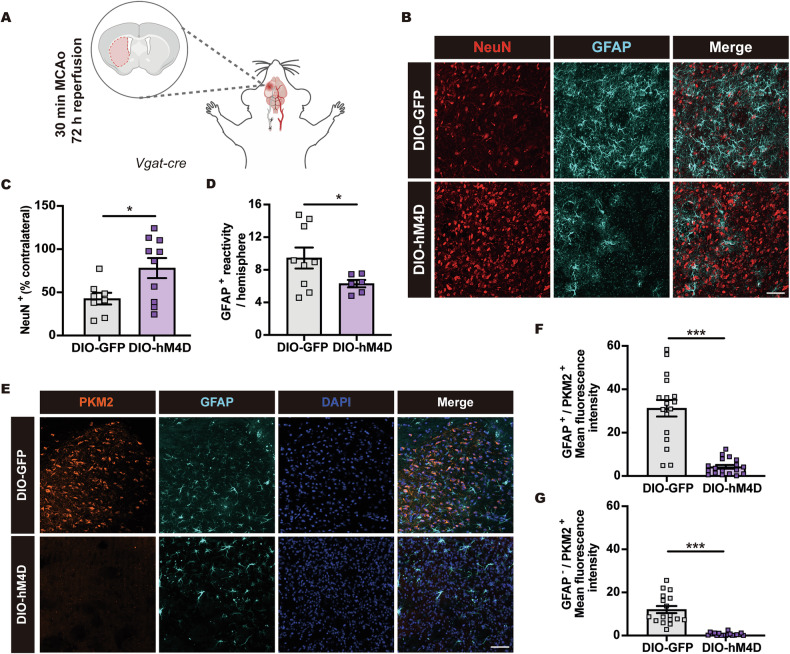
DMH^VGAT^ silencing increases post-ischemic neuronal survival and preserves the homeostatic astrocyte state. **A** Cartoon depicting the 30 min MCAo model followed by 72 h reperfusion that results in disseminate neuronal injury in the striatum. **B** Representative photomicrographs showing the effect of CLZ-induced DMH^VGAT^ silencing on neuronal survival and astroglial responses evaluated by NeuN and GFAP immunolabeling. Quantification of **C** neuronal survival and **D** astroglial GFAP immunoreactivity in DMH^VGAT:hM4D^ mice (*n* = 10) compared to DMH^VGAT:GFP^ control mice (*n* = 9). **E** Representative photomicrographs and **F**, **G** quantification of PKM2 expression, a marker of the detrimental “neurotoxic” astrocyte state, in GFAP^+^ astrocytes and GFAP^−^ cells (cell nuclei counterstained with DAPI). Note that CLZ-induced DMH^VGAT^ silencing reduced astrocytic PKM2 expression, indicative that astrocytes retained their homeostatic phenotype (*n* = 17–18 ROIs in *n* = 4 mice per group). Scale bars: 50 µm. Data are mean ± SEM values. *p < 0.05, ***p < 0.001.

We have previously shown that disseminate neuronal injury can be detected in the ischemic striatum when mice are exposed to 30 min MCAo [34, 36]. Earlier studies revealed that this type of cell injury evolves over up to 72 h post-MCAo and that it mostly affects small- to medium-sized interneurons [37, 38]. Therefore, we next investigated whether hypothermia induced by chemogenetic DMH^VGAT^ silencing increases neuronal survival in the striatum of 30 min MCAo mice (Fig. 4A and Supplementary Fig. S1B). Indeed, DMH^VGAT^ silencing promoted neuronal survival in this mild ischemic brain injury model (Fig. 4B, C and Supplementary Fig. S2A). Thus, the percentage of surviving NeuN^+^ neurons was increased in DMH^VGAT^ silenced DIO-hM4D compared to non-silenced DIO-GFP mice (Fig. 4B, C).

Astrocytes support brain homeostasis under physiological conditions, but exhibit inflammatory changes post-ischemia associated with GFAP immunoreactivity [39, 40], which provides the basis for subsequent glial scar development [41]. We thus investigated the impact of chemogenetic DMH^VGAT^ silencing on astrocytic GFAP responses following 30 min MCAo, showing that DMH^VGAT^ silencing decreased the area of brain tissue covered by GFAP^+^ reactive astrocytes (Fig. 4B, D).

Under homeostatic conditions, astrocytes take up glutamate from the extracellular space and detoxify the brain tissue [42]. Upon ischemia, astrocytes may adopt a neurotoxic state characterized by compromised mitochondrial function that is predominated by lactate-directed glycolysis [43]. PKM2, which regulates mitochondrial function [44], plays a central role in the metabolic switch of homeostatic astrocytes to neurotoxic astrocytes [43]. Given that PKM2 is upregulated upon ischemia [45], we investigated the expression of PKM2 in GFAP^+^ astrocytes. Indeed, astrocytes exhibited robust cytosolic PKM2 expression post-ischemia, and PKM2 levels markedly decreased in response to hypothermia induced by DMH^VGAT^ silencing (Fig. 4E–G). Notably, this reduction was not restricted to GFAP^+^ astrocytes but was also evident in GFAP^-^ cell populations, which presumably involved non-astrocytic cell populations (Fig. 4E–G). In addition to its anti-apoptotic role, Bcl-xL maintains mitochondrial function in astrocytes [46]. We next investigated whether hypothermia induced by DMH^VGAT^ silencing increases Bcl-xL levels in the ischemic brain. Bcl-xL and GFAP immunostaining confirmed that Bcl-xL levels increased upon DMH^VGAT^ silencing, but only modestly in astrocytes (Supplementary Fig. S2A, B). Taken together, our findings indicate that hypothermia induced by chemogenetic DMH^VGAT^ silencing promotes neuronal survival by preservation of the homeostatic astrocyte state.

### DMH^VGAT^ silencing reduces post-ischemic reperfusion and decreases reperfusion injury

**Fig. 5:**
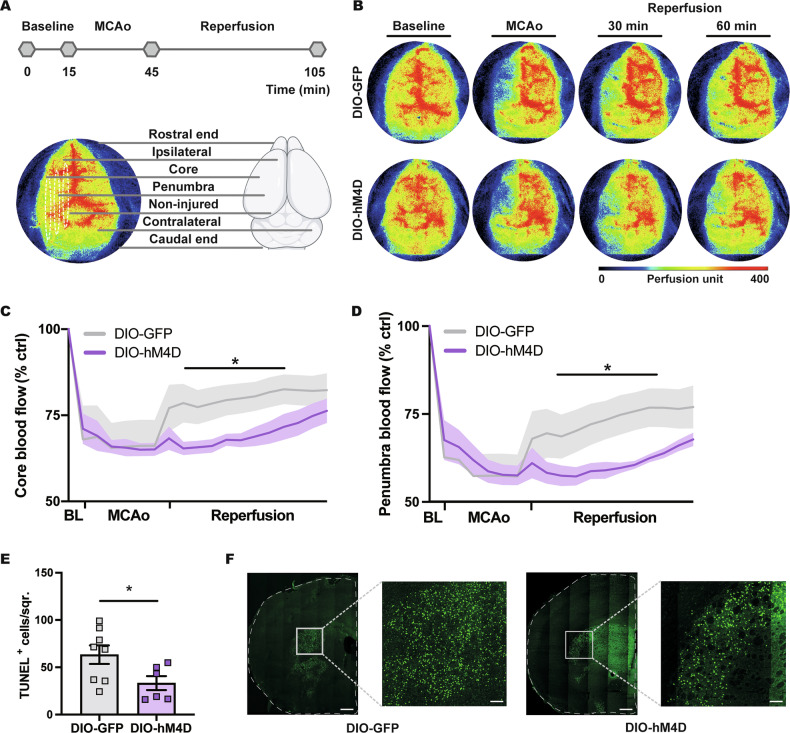
DMH^VGAT^ silencing decreases post-ischemic reperfusion and reperfusion injury. **A** Experimental timeline and areas of interest for analysis of laser speckle images after chemogenetic DMH^VGAT^ silencing. **B** Representative laser speckle images in response to CLZ in DMH^VGAT:hM4D^ and DMH^VGAT:GFP^ mice at baseline, during MCAo and at 30- and 60-min following reperfusion. Quantitative analysis of regional CBF in **C** the ischemic core and **D** the penumbra showing decreased reperfusion after CLZ-induced DMH^VGAT^ silencing (*n* = 3 each). **E** Effect of chemogenetic DMH^VGAT^ silencing on post-ischemic reperfusion injury in the striatum assessed by TUNEL in DMH^VGAT:hM4D^ (*n* = 10) and DMH^VGAT:GFP^ control mice (*n* = 9). In **F**, representative TUNEL stainings exhibiting DNA-fragmented cells are shown. Note the strong reduction of TUNEL^+^ cells in the striatum of DMH^VGAT:hM4D^ compared to DMH^VGAT:GFP^ control mice. Scale bars: 1 mm (overview images)/250 µm (magnifications). Data are mean ± SEM values. *p < 0.05.

Vasoconstriction is one of the most critical responses, via which mammals cope with hypothermia [47]. Considering that vasoconstriction-associated CBF reduction may protect the brain against neuronal injury by reducing oxidative stress [48, 49], we next examined if chemogenetic DMH^VGAT^ silencing influenced post-ischemic reperfusion. To test this, laser speckle imaging was performed in *Vgat-cre* mice. Regional CBF was monitored during 30 min MCAo followed by 60 min reperfusion (Fig. 5A). Our findings revealed that the chemogenetic deactivation of DMH^VGAT^ neurons potently decreased regional CBF during reperfusion in the ischemic core and penumbra regions in DIO-hM4D compared to DIO-GFP mice (Fig. 5B–D). Of note, regional CBF very slowly recovered over the 1-h-interval post-ischemia in hypothermic DMH^VGAT^ silenced mice. We conclude that chemogenetically induced hypothermia protects against ischemia by preventing cerebral reperfusion injury.

To elucidate how chemogenetic DMH^VGAT^ silencing influences reperfusion injury in the previously ischemic striatum, we subsequently evaluated irreversible cell injury by DNA fragmentation analysis. Our findings revealed that chemogenetic deactivation of DMH^VGAT^ neurons decreased the number of DNA fragmented cells indicative of reduced reperfusion injury (Fig. 5E, F).

### Optogenetic activation of the GABAergic vlPOA^VGAT^ → DMH pathway induces hypothermia

**Fig. 6:**
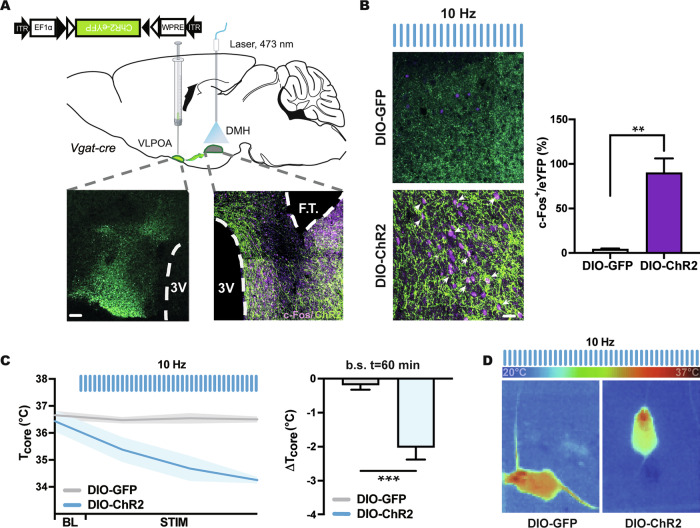
Optogenetic activation of GABAergic vlPOA^VGAT^ → DMH pathway induces hypothermia. **A** Cartoon illustrating optogenetic activation of the vlPOA^VGAT^ → DMH pathway as a strategy for inducing hypothermia. An AAV vector with double-floxed inverted open-reading frame (DIO) hChR2-eYFP was unilaterally injected into the vlPOA of *Vgat-cre* mice, resulting in selective expression of hChR2-eYFP in GABAergic vlPOA neurons and DMH-projecting nerve fibers, as shown in representative microscopic images. Representative photomicrograph showing fiber tip (F.T.) placement above the DMH. Scale: 500 μm. Optical stimulation was performed with 473 nm laser light. **B** Photomicrograph (left) and quantification (right) of c-Fos expression in the DMH of ChR2-expressing (*n* = 36) or GFP-expressing (*n* = 4) mice during 10 Hz photostimulation. **C** Effect of optical stimulation from the fiber tip on core body temperature in vlPOA^VGAT:ChR2^ mice (*n* = 6) compared to vlPOA^VGAT:GFP^ control mice (*n* = 8). Maximum body temperature changes were observed at *t* = 60 min. **D** Infrared thermography demonstrating decreased body temperature and tail vasoconstriction upon optical stimulation of vlPOA^VGAT^ nerve terminals in the DMH. Scale bar: 50 µm. Data are mean ± SEM values. ***p < 0.001.

Previous studies demonstrated that DMH receives dense GABAergic inputs from the vlPOA, which contribute to hypothalamic core body temperature regulation [26]. To gain insight into the role of DMH-projecting vlPOA^VGAT^ neurons in hypothermia induction, we transduced GABAergic neurons located in the vlPOA of *Vgat-cre* mice by administering a Cre-dependent *rAAV2/1-EF1a-DIO-hChR2(H134R)-EYFP-WPRE-HGHpA* vector. Transduction of vlPOA^VGAT^ neurons by DIO-hChR2 yielded robust axonal labeling in the DMH (Fig. 6A). Optogenetic activation of vlPOA^VGAT^ → DMH neurons induced the co-expression of eYFP and c-Fos in vlPOA^VGAT^ synapses of DIO-hChR2 mice (Fig. 6B). We subsequently investigated the role of the vlPOA^VGAT^ → DMH projection in core body temperature regulation by stimulating vlPOA^VGAT^ terminals in the DMH using 473 nm laser light pulses. Here, we found that optical stimulation significantly reduced *T*_core_ by 2.02 ± 0.34 °C (Fig. 6C, D), which suggested that activation of vlPOA^VGAT^ terminals recapitulated the effect of DMH^VGAT^ silencing. Upon careful inspection, we did not observe any behavioral abnormalities upon optogenetic vlPOA^VGAT^ activation, emphasizing the specificity of the temperature response. Collectively, the functional findings obtained indicated that vlPOA^VGAT^ neurons induce hypothermia by inhibiting DMH^VGAT^ neurons.

### vlPOA^VGAT^ → DMH pathway activation induces post-ischemic neuroprotection and preserves astrocytic homeostasis

**Fig. 7:**
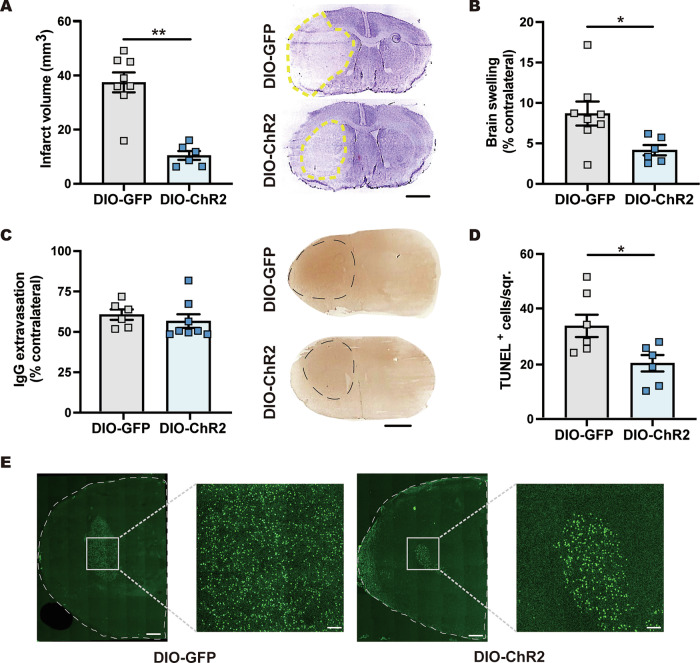
Activation of vlPOA^VGAT^ → DMH pathway reduces ischemic infarct volume, brain edema and cell injury. Effect of optogenetic activation of the vlPOA^VGAT^ → DMH pathway on **A** infarct volume and **B** brain edema examined by cresyl violet staining as well as **C** BBB leakage assessed by IgG extravasation analysis in optogenetically stimulated vlPOA^VGAT:ChR2^ mice (*n* = 6) compared to vlPOA^VGAT:GFP^ control mice (*n* = 8) exposed to 90 min MCAo followed by 24 h reperfusion. Representative cresyl violet and IgG stainings are shown. Scale bars: 2 mm. **D** Effect of optogenetic vlPOA^VGAT^ → DMH projection activation on post-ischemic reperfusion injury in the striatum assessed by TUNEL in vlPOA^VGAT:ChR2^ and vlPOA^VGAT:GFP^ mice (*n* = 7 each) exposed to 30 min MCAo followed by 72 h reperfusion. In **E**, representative TUNEL stainings exhibiting DNA-fragmented cells are shown. Note the strong reduction of TUNEL^+^ cells in the striatum of vlPOA^VGAT:ChR2^ compared with vlPOA^VGAT:GFP^ control mice. Scale bars: 1 mm (overview images)/250 µm (magnifications). Data are mean ± SEM values. *p < 0.05, **p < 0.01.

Given the involvement of DMH-projecting vlPOA^VGAT^ neurons in inducing hypothermia, we further asked whether activation of this pathway is able to phenocopy the effects of chemogenetic DMH^VGAT^ silencing on ischemic brain tissue survival. For this, we again induced 90 min occlusion to introduce focal infarcts (Supplementary Fig. S1C). Optogenetic activation of the vlPOA^VGAT^ → DMH projection reduced ischemic injury at 24 h post-MCAo. Indeed, infarct volume and brain edema were significantly reduced by vlPOA^VGAT^ → DMH activation (Fig. 7A, B), whereas BBB leakage evaluated by IgG extravasation was modestly, but non-significantly decreased (Fig. 7C). Taken together, activating the nerve terminals of DMH-projecting vlPOA^VGAT^ neurons mimics the effects of DMH^VGAT^ silencing on ischemic brain injury.

**Fig. 8:**
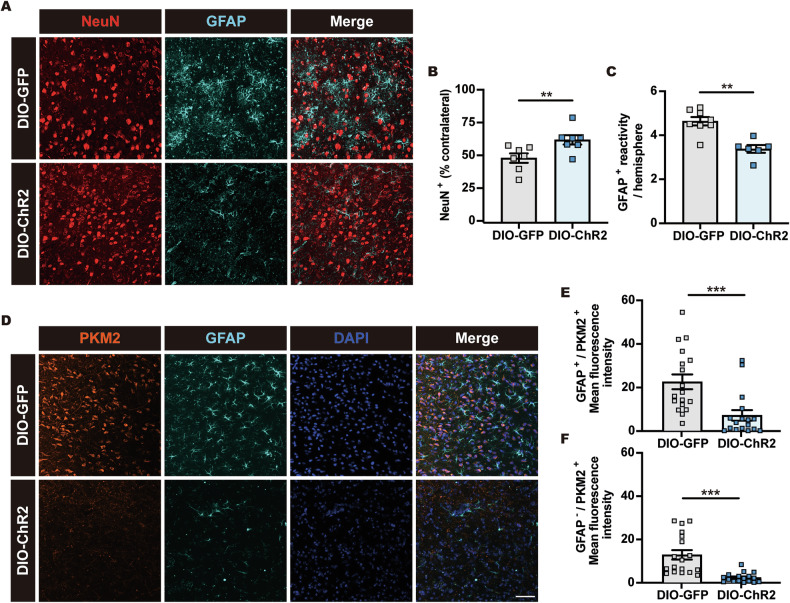
vlPOA^VGAT^ → DMH pathway activation increases post-ischemic neuronal survival and preserves astrocytic homeostasis. **A** Representative photomicrographs showing the effect of optogenetic vlPOA^VGAT^ → DMH pathway activation on neuronal survival and astroglial responses evaluated by NeuN and GFAP immunolabeling in mice exposed to 30 min MCAo followed by 72 h reperfusion. Quantification of **B** neuronal survival and **C** astroglial GFAP immunoreactivity in stimulated vlPOA^VGAT:ChR2^ mice (*n* = 7) compared to vlPOA^VGAT:GFP^ control mice (*n* = 7). **D** Representative photomicrographs and **E**, **F** quantification of PKM2 immunoreactivity, a marker of “neurotoxic” astrocytes, in GFAP^+^ astrocytes and GFAP^-^ cells (nuclei counterstained with DAPI). Note that optogenetic vlPOA^VGAT^ → DMH activation reduced PKM2 expression, indicative that astrocytes retained their homeostatic state (*n* = 17–18 ROIs in *n* = 4 mice per group). Scale bars: 50 µm. Data are mean ± SEM values. **p < 0.01, ***p < 0.001.

We next investigated whether optogenetic activation of the vlPOA^VGAT^ → DMH pathway similarly provided neuroprotection following mild ischemic injury induced by 30 min MCAo (Supplementary Fig. S1D). Indeed, we observed decreased disseminate cell injury assessed by TUNEL (Fig. 7D, E). NeuN immunostaining revealed that optogenetic activation of vlPOA^VGAT^ → DMH projecting nerve terminals increased neuronal survival in the striatum 72 h after 30 min MCAo (Fig. 8A, B). Optogenetically induced hypothermia reduced post-ischemic astrocyte reactivity and neurotoxicity, as indicated by the downregulated GFAP and PKM2 expression (Fig. 8A, C–E). Additionally, we observed decreased expression of PKM2 and Bcl-xL in GFAP^-^ cells (Fig. 8F and Supplementary Fig. S2C, D), suggesting adaptive mitochondrial regulation in non-astrocytic cells. These findings indicate that hypothermia induced by optogenetic vlPOA^VGAT^ → DMH pathway activation preserves the homeostatic role of astrocytes and promotes neuronal survival after stroke.

## Discussion

Multiple lines of evidence suggest that mild hypothermia potently promotes neuronal survival and prevents cell death in experimental brain injury, including ischemic stroke models [50–54]. So far, therapeutic hypothermia induced through systemic and intra-arterial cooling failed to enhance neurological outcome in ischemic stroke patients in randomized clinical trials, which was due to multiple side-effects, namely cardiac rhythm disturbances, hypokalemia, coagulopathy and infections, which compromised stroke recovery [21–25, 55–57]. In view of the persisting deficits of stroke patients in the post-acute stroke phase, there is a need for treatments that enhance stroke recovery. Given the strong neuroprotective effect induced by the activation of the GABAergic vlPOA^VGAT^ pathway to the DMH, this pathway might provide a promising target for treatments that enhance stroke outcome. Indeed, optogenetic activation of vlPOA^VGAT^ neurons potently induced hypothermia in *Vgat-cre* mice, as did chemogenetic silencing of DMH^VGAT^ neurons, both of which induced neuroprotection by inhibiting cerebral reperfusion injury.

The maintenance of body temperature is critical for cell survival and homeostasis. Body temperature regulation is strictly orchestrated by thermoregulatory networks in the anterior and dorsomedial hypothalamus [26, 58], which integrate peripheral thermal, endocrine, autonomic and behavioral information [3, 4]. Involvement of the DMH in thermal information processing has been suggested in studies showing that DMH^VGAT^ neurons induced thermogenesis when activated chemogenetically [26]. Conversely, optogenetic silencing of DMH^VGAT^ neurons was found to induce hypothermia [26]. Consistent with earlier studies, our study showed that chemogenetic DMH^VGAT^ silencing potently decreases *T*_core_, which goes in line with reduced physical activity. In contrast to our study and the above earlier studies, a recent study did not observe *T*_core_ changes when GABAergic DMH neurons were optogenetically activated over a shorter time interval [59]. In view of this apparent dependency on the duration and timing of the therapeutic intervention, we initiated chemogenetic DMH^VGAT^ silencing before MCAo and retained it until the end of the occlusion to ensure a significant loss of body temperature.

Warm-sensitive neurons in the preoptic area are a key sensor and regulator of thermal homeostasis [60, 61] that project to distinct effector regions in the brain to regulate brown adipose tissue activity and skin vasodilation [26, 58]. The preoptic area integrates inputs from ascending thermoregulatory pathways in the brainstem and spinal cord that convey information from the body periphery [62, 63]. Interestingly, tracing studies showed that the vlPOA provides a significant projection to the DMH [64, 65]. GABAergic neurons in the vlPOA were shown to evoke tonic inhibition of brown adipose tissue activity via the DMH [66, 67], whereas pharmacological inhibition of GABA_A_ receptors in the DMH stimulated brown adipose tissue activity by sympathetic activation [68, 69]. Previously, GABAergic neurons located at the vlPOA were found to suppress cold-activated DMH ^VGAT^ neurons to lower core body temperature [26]. Consistent with this study, we demonstrated that the DMH receives robust input from vlPOA^VGAT^ neurons. We found that optogenetic activation of vlPOA^VGAT^ originating nerve terminals in the DMH similarly as chemogenetic DMH^VGAT^ silencing potently induced hypothermia.

Our study shows that chemogenetic DMH^VGAT^ neuron silencing and optogenetic activation of GABAergic vlPOA^VGAT^ fibers terminating in the DMH induced hypothermia in *Vgat-cre* mice to reduce infarct volume, brain edema, neurological deficits, reactive astroglial responses and cerebral reperfusion injury. Upon careful inspection, we did not observe any behavioral abnormalities associated with chemogenetic and optogenetic interventions in this study. Therapeutic hypothermia induced by animal cooling potently has previously been shown to decrease ischemic injury in experimental stroke models by regulating CBF, decreasing oxidative stress and inflammation, and preserving BBB integrity [8–12]. Our data indicate that hypothermia induced by the optogenetic activation of the GABAergic vlPOA^VGAT^ → DMH pathway recapitulates these effects, as does chemogenetic DMH^VGAT^ silencing.

Astrocytes are key for homeostatic responses to brain injury [42]. After a stroke, astrocytes undergo inflammatory changes associated with GFAP immunoreactivity [39, 40], which provides the basis for subsequent glial scar development [41]. Reprogramming in ischemic astrocytes can be both beneficial (neuroprotective) and detrimental (neurotoxic) [39, 40, 70]. Previously, activation of the PKM2-STAT3-hypoxia-inducible factor (HIF) pathway has been described as a key trigger of astrocytic neurotoxicity after stroke [43]. Metabolic reprogramming in ischemic astrocytes induces PKM2-mediated phosphorylation and activation of transcriptional factor STAT3, which in turn induces HIF expression. Activation of hypoxia-responsive elements for HIF promotes PKM2 expression, creating the “vicious cycle” of neurotoxic astrocytic remodeling. In this study, we demonstrate that hypothermia induced by both chemogenetic DMH^VGAT^ silencing and optogenetic vlPOA^VGAT^ → DMH activation downregulates PKM2 expression in neurotoxic astrocytes, contributing to post-ischemic brain tissue survival.

Cold exposure has long been known to induce vasoconstriction [47, 71]. Two aspects explain the lowering of blood flow in hypothermia, which is the need of counteracting heat loss through the body surface and the reduction of cell metabolism requiring less oxygen and energy-rich substrates to be provided via the blood. In the present study, we found markedly reduced post-ischemic regional CBF in the brains of mice exhibiting chemogenetic DMH^VGAT^ deactivation. The reduction of energy consumption post-ischemia protects the brain, since it reduces cerebral cellular reperfusion injury. Of note, we indeed observed reduced disseminate cell death in the brains of mice receiving chemogenetic DMH^VGAT^ silencing and optogenetic vlPOA^VGAT^ → DMH activation, which supports this mechanism. That tissue reperfusion in animals undergoing transient focal cerebral ischemia may augment brain injury above levels observed in permanent focal cerebral ischemia is well established [72, 73]. Our data suggest that therapeutic hypothermia induced by DMH^VGAT^ deactivation and vlPOA^VGAT^ → DMH activation counteracts the post-ischemic reperfusion injury, enabling successful recovery of the brain tissue.

A limitation of our study is the initiation of hypothermia on the occasion of the stroke onset. However, we did not directly test the effect of post-ischemic hypothermia induced by chemogenetic DMH^VGAT^ silencing and optogenetic vlPOA^VGAT^ → DMH activation. Future experiments will have to define therapeutic windows of chemogenetically and optogenetically induced neuroprotection. The model used is a clinically relevant model of post-ischemic reperfusion damage, which following recent advances in reperfusion therapies (thrombolysis combined with mechanical thrombectomy) has gained clinical importance. We did not test the effect of hypothermia induced by DMH^VGAT^ neuron silencing and vlPOA^VGAT^ → DMH pathway activation on female animals and aged animals with vascular risk factors or comorbidities. Future studies will have to elucidate long-term outcomes in mice with chemogenetically or optogenetically induced hypothermia.

In summary, we herein identified a GABAergic vlPOA^VGAT^ → DMH pathway, which mediates potent neuroprotection in mice exposed to transient MCAo. In view of the well-defined temperature response, this projection may provide a potent target for neuroprotection not only in ischemic stroke, but also in other brain injury conditions, including cardiac arrest, traumatic brain injury, subarachnoid hemorrhage or intracerebral hemorrhage. Transcranial magnetic stimulation techniques have made huge progress in recent years, and it is meanwhile possible to stimulate deep brain structures in a localized way [74]. We predict that via stimulation of the vlPOA^VGAT^ → DMH pathway, it might be possible to enhance clinical outcome of brain-injured patients. Since this procedure does not involve exogenous peripheral or intra-arterial cooling, this treatment has the potential of bypassing the side effects previously noted in randomized controlled hypothermia trials, in which cardiac rhythm disturbances, hypokalemia, coagulopathy and infections compromised clinical outcome of ischemic stroke patients [21–25]. Experimental studies are warranted on this topic.

## Supplementary information

Supplementary legends

Supplementary Table 1

Supplementary Fig. S1

Supplementary Fig. S2

## Data Availability

The datasets generated and analyzed during the current study are available from the corresponding author upon reasonable request.
