# Supplementary material for: GABAergic ventrolateral preoptic projection to dorsomedial hypothalamus recapitulates post-ischemic neuroprotection by hypothermia
Source: Cell Death Dis. 2026 Mar 10;17(1):304. doi: 10.1038/s41419-026-08536-0 (PMC13039838; doi:10.1038/s41419-026-08536-0)
Supplement: Supplementary file 1 — Supplementary legends [file 41419_2026_8536_MOESM1_ESM.docx]

**Supplementary legends:**

**Supplementary Fig. S1: Cerebral blood flow recordings by Laser Doppler flow measurement.** Cerebral blood flow (CBF) was measured by Laser Doppler flow measurement above the core of the middle cerebral artery territory during and after 90 min **(A, C)** or 30 min **(B, D)** intraluminal middle cerebral artery occlusion (MCAo) in mice exposed to chemogenetically or optogenetically induced hypothermia and their control mice (n=6-10 mice per group). Data are mean ± SEM values.

**Supplementary Fig. S2: Chemogenetic DMH^VGAT^ silencing and optogenetic vlPOA^VGAT^ 🡪 DMH pathway activation increase post-ischemic Bcl-xL expression mainly in non-astrocytic cells.** **(A, C)** Representative immunolabeling of the mitochondrial protein Bcl-xL and the astrocytic marker protein GFAP in the ischemic striatum of MCAO mice exposed to chemogenetic DMH^VGAT^ silencing (in **A**) or optogenetic vlPOA^VGAT^ 🡪 DMH pathway activation (in **C**). **(B, D)** Quantification of Bcl-xL expression in GFAP^+^ astrocytes and GFAP^-^ non-astrocytic cells under conditions of chemogenetic DMH^VGAT^ silencing (in **B**) or optogenetic vlPOA^VGAT^ 🡪 DMH pathway activation (in **D**) (n=17-18 ROIs in n=4 mice per group). Scale bars: 50 µm. Data are mean ± SEM values. ***p < 0.001.

**Supplementary Table 1: Statistical table.** Statistical table summarizing the data structure, type of test applied, p-values and power of the findings.
