# Supplementary material for: GABAergic ventrolateral preoptic projection to dorsomedial hypothalamus recapitulates post-ischemic neuroprotection by hypothermia
Source: Cell Death Dis. 2026 Mar 10;17(1):304. doi: 10.1038/s41419-026-08536-0 (PMC13039838; doi:10.1038/s41419-026-08536-0)
Supplement: Supplementary file 2 — Supplementary Table 1 [file 41419_2026_8536_MOESM2_ESM.docx]

**Supplementary Table 1: Statistical Table**

| **Graph** | **Data structure** | **Type of test** | **p-values** | **Power (95% C.I. of diff)** |
| --- | --- | --- | --- | --- |
| Figure 2C | Normal distribution, non-parametric | Mann-Whitney U test | 0.0022 | -2.500 to -1.000 |
| Figure 2E | Normal distribution, non-parametric | Mann-Whitney U test | 0.0024 | -0.09000 to -0.03100 |
| Figure 2F | Non-normal distribution, non-parametric | Mann-Whitney U test | 0.0028 | -254.0 to -87.90 |
| Figure 2G | Normal distribution, non-parametric | Mann-Whitney U test | 0.0028 | -53.13 to -21.94 |
| Figure 3B | Non-normal distribution, non-parametric | Mann-Whitney U test | 0.0027 | -35.18 to -3.410 |
| Figure 3C | Normal distribution, non-parametric | Mann-Whitney U test | 0.2284 | -14.45 to 3.420 |
| Figure 3D | Normal distribution, non-parametric | Mann-Whitney U test | 0.1812 | -61.75 to 7.303 |
| Figure 3E | Non-normal distribution, non-parametric | Mann-Whitney U test | 0.0213 | -1.500 to 0.000 |
| Figure 4C | Normal distribution, parametric | Unpaired Student’s *t* test  (Two-tailed P value) | 0.0193 | 6.680 to 64.24 |
| Figure 4D | Normal distribution, non-parametric | Mann-Whitney U test | 0.0443 | -6.186 to -0.09719 |
| Figure 4F | Normal distribution, parametric | Unpaired Student’s *t* test  (Two-tailed P value) | <0.0001 | -35.23 to -18.80 |
| Figure 4G | Normal distribution, parametric | Unpaired Student’s *t* test  (Two-tailed P value) | <0.0001 | -14.94 to -7.890 |
| Figure 5E | Normal distribution, non-parametric | Mann-Whitney U test | 0.0308 | -56.89 to -3.289 |
| Figure 6B | Normal distribution, non-parametric | Mann-Whitney U test | 0.0095 | 37.00 to 139.0 |
| Figure 6C | Non-normal distribution, non-parametric | Mann-Whitney U test | <0.0001 | 1.000 to 2.400 |
| Figure 7A | Normal distribution, non-parametric | Mann-Whitney U test | 0.0013 | -36.96 to -19.93 |
| Figure 7B | Normal distribution, non-parametric | Mann-Whitney U test | 0.0200 | -7.359 to -1.176 |
| Figure 7C | Non-normal distribution, non-parametric | Mann-Whitney U test | 0.1682 | -30.00 to 18.23 |
| Figure 7D | Normal distribution, non-parametric | Mann-Whitney U test | 0.0350 | -26.43 to -0.07000 |
| Figure 8B | Normal distribution, non-parametric | Mann-Whitney U test | 0.0082 | 2.229 to 26.29 |
| Figure 8C | Normal distribution, non-parametric | Mann-Whitney U test | 0.0013 | -1.831 to -0.7291 |
| Figure 8E | Non-normal distribution, parametric | Unpaired Student’s *t* test  (Two-tailed P value) | 0.0009 | -23.91 to -6.889 |
| Figure 8F | Non-normal distribution, parametric | Unpaired Student’s *t* test  (Two-tailed P value) | 0.0002 | -15.27 to -5.849 |
| Supplementary Figure S2B (upper) | Non-normal distribution, parametric | Unpaired Student’s *t* test  (Two-tailed P value) | 0.3788 | -7.900 to 20.27 |
| Supplementary Figure S2B (lower) | Non-normal distribution, parametric | Unpaired Student’s *t* test  (Two-tailed P value) | <0.0001 | 3.056 to 8.208 |
| Supplementary Figure S2D (upper) | Non-normal distribution, parametric | Unpaired Student’s *t* test  (Two-tailed P value) | 0.1991 | -15.06 to 17.90 |
| Supplementary Figure S2D (upper) | Normal distribution, parametric | Unpaired Student’s *t* test  (Two-tailed P value) | <0.0001 | 6.077 to 14.09 |
