# Supplementary figures and images for: GABAergic ventrolateral preoptic projection to dorsomedial hypothalamus recapitulates post-ischemic neuroprotection by hypothermia
Source: Cell Death Dis. 2026 Mar 10;17(1):304. doi: 10.1038/s41419-026-08536-0 (PMC13039838; doi:10.1038/s41419-026-08536-0)

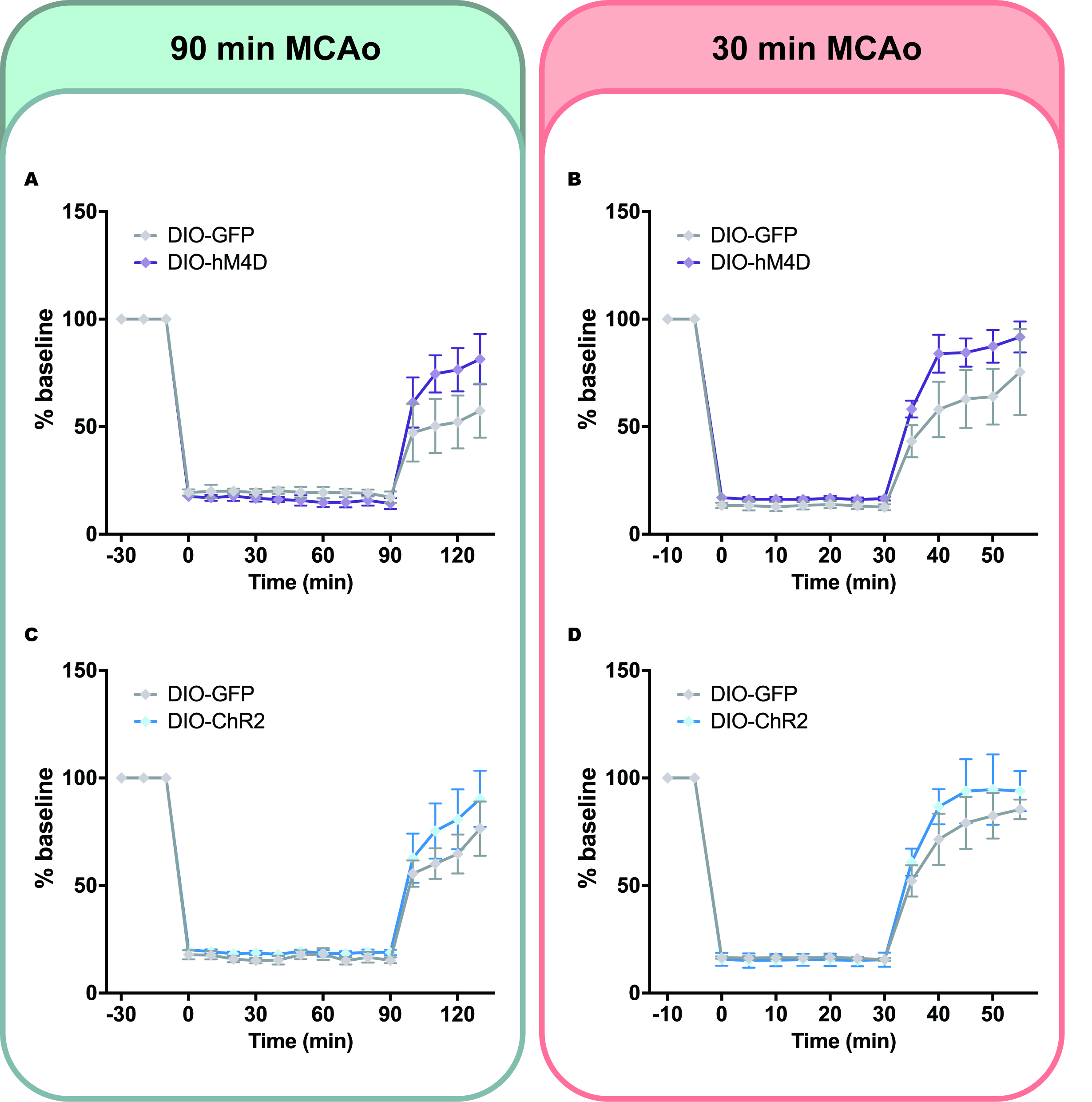

Supplement: Supplementary file 3 — Supplementary Fig. S1 [file 41419_2026_8536_MOESM3_ESM.tif]

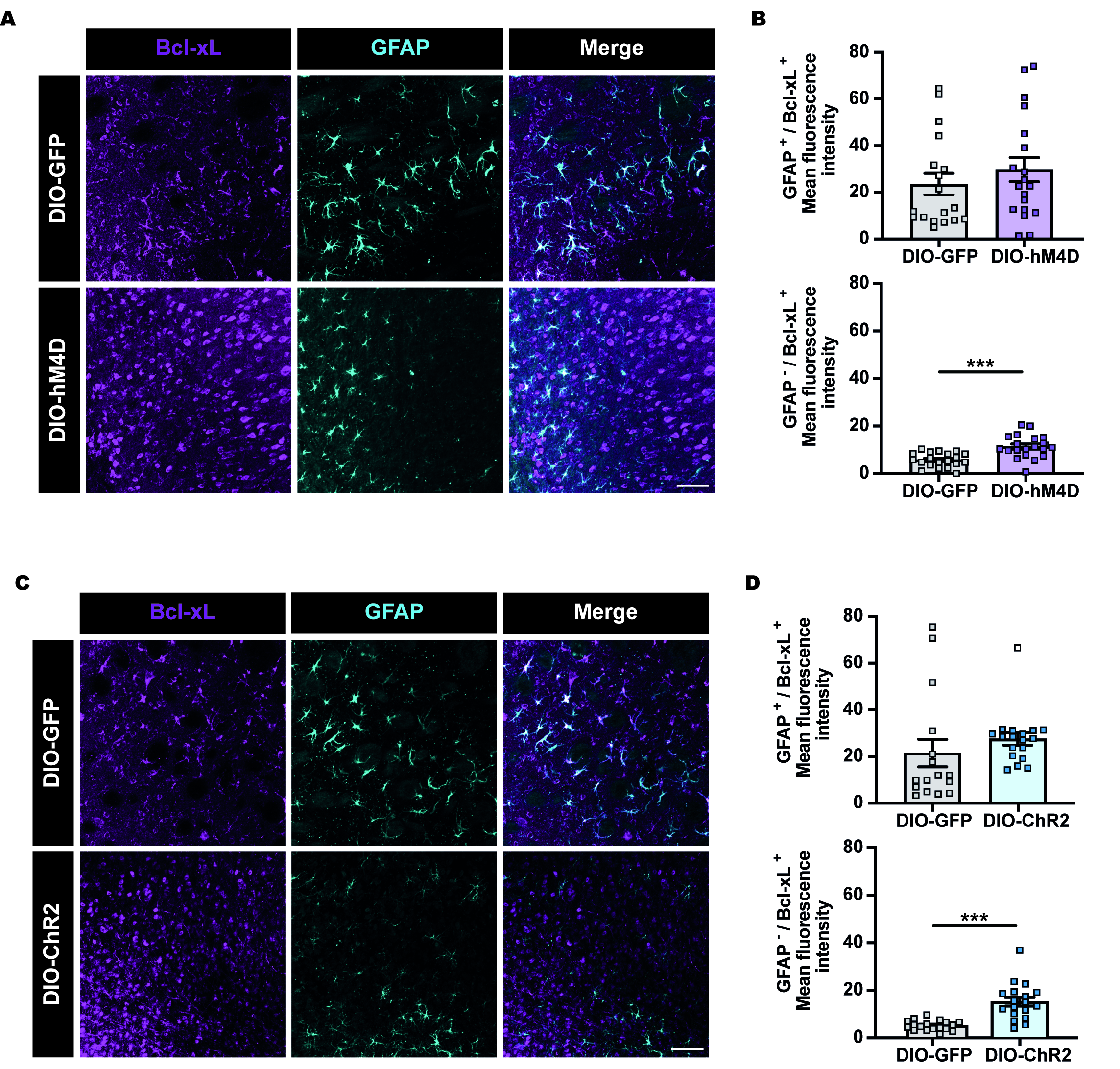

Supplement: Supplementary file 4 — Supplementary Fig. S2 [file 41419_2026_8536_MOESM4_ESM.tif]
